# Molecular Dynamics Study on the Effect of Surface Hydroxyl Groups on Three-Phase Wettability in Oil-Water-Graphite Systems

**DOI:** 10.3390/polym9080370

**Published:** 2017-08-18

**Authors:** Wenxiu Zheng, Chengzhen Sun, Bofeng Bai

**Affiliations:** State Key Laboratory of Multiphase Flow in Power Engineering, Xi’an Jiaotong University, Xi’an 710049, China; zhengwenxiu1989@stu.xjtu.edu.cn (W.Z.); sun-cz@mail.xjtu.edu.cn (C.S.)

**Keywords:** hydroxyl, graphite surface, wettability, hydrogen bond

## Abstract

In this paper, a hydroxylated graphite surface is generated as a hydrophilic oleophobic material for the application of oil-water separation, and the effects of hydroxyl density on the three-phase wettability are studied in oil-water-graphite systems. We analyze the adsorption of water molecules on the hydroxylated surfaces and obtain the relationship between water-oil-solid interfacial properties and the hydroxyl density, which results from the synthetic effects of the orientation of molecules and hydrogen bonds. With the increase of hydroxyl density, the water-solid contact angle first decreases rapidly, and then remains constant. The density of the hydrogen bond formed between hydroxyls and water molecules in the adsorption layer can explain the regularity of the three-phase wettability. The orientation of the water molecules in the adsorption layer shows insignificant variation, owing to the hydrogen bond network formed between the water molecules; thus, little change is observed in the hydrogen bond density in the adsorption layer.

## 1. Introduction

The water content of crude oil increases during oil recovery due to water injection, leading to increases attention to the study of oil-water separation [1]. There are several types of membrane material that are often used in oil-water separation, including hydrophobic oleophilic materials (oil passes through the membrane) [2] and hydrophilic oleophobic materials (water passes through the membrane) [3]. Researchers [4] have found that hydrophilic and oleophobic molecules can be assembled together on a membrane surface, which shows good separation qualities. Furthermore, hydrophilic oleophobic materials are not easily fouled by oil, and thus can be recycled easily [5]. Thus, many researchers pay more attention to hydrophilic oleophobic materials. The main idea is to prepare a hydrophilic oleophobic surface in a water environment [6]. For this process, the wettability problem [7] in the oil-water-solid three-phase system needs to be studied. For the three-phase system, the wettability problem becomes more complex due to the combination of both oil and water bulk. Xue et al [8] tested the wettability of nano-roughness modified surfaces, which showed oleophobic properties in water. The mechanism of the changing wettability is related to the ordering of molecules near the wall [9].

Carbon materials have significant applications in many industries, such as oil-water separation, membrane separation, micro-electronics and so on [10,11,12]. Graphite is a typical example of a carbon material that has many advantages, such as high stability, corrosion resistance to acid/alkali and organic solvents, and the ability to withstand severe changes of temperature [13,14]. It is therefore often used as the hydrophobic oleophilic material for the oil-water separation. Hydrophilic oleophobic surfaces made out of graphite have the advantages of both graphite and hydrophilic surfaces. Hydroxyl is a common hydrophilic group, and can be used to modify the surface and change the wettability, owing to the influence of hydrogen bonds [15]. Emami et al. showed that H_2_O on a silica surface with different hydroxyl density exhibited different behaviors. The surface is entirely wetted by H_2_O for high hydroxyl density [16]. Eslami et al. displayed that hydrogen bond formation shows different rule with changes of surface curvature, which is due to the greater penetration over more curved surfaces [17]. Matheus et al. showed an increase in the ability of ion liquids to accept hydrogen bonds on a more polar surface (i.e., with lower contact angles) [18]. However, the ordering of molecules at the surface may impose limitations on the change of wettability with an increase in hydroxyl density. Meanwhile, water molecules can form a hydrogen bond network, which may result in a more stable structure [19], and which may have a negative effect on the formation of the hydrogen bonds between water molecules and hydroxyls. With the increase of hydroxyl density, the effects of hydroxyls on the orientation of water molecules and the density of hydrogen bonds in the adsorption layer may be different, contributing significantly to the surface wettability.

In this paper, we focus on generating a hydroxylated graphite surface, and reveal the mechanism of the effect of hydroxyls on the wettability of graphite surfaces in a three-phase system using the Molecular Dynamics (MD) simulation. In the next section, the details of the MD model are illustrated, including the interatomic interaction potential and the verification of the basic method. Results of molecule configurations and microscopic contact angle in systems with different hydroxyl densities are then shown. Additionally, the adsorption properties of water molecules and the number of hydrogen bonds are obtained. The final section provides a summary of the results.

## 2. Model and Methods 

**Figure 1:**
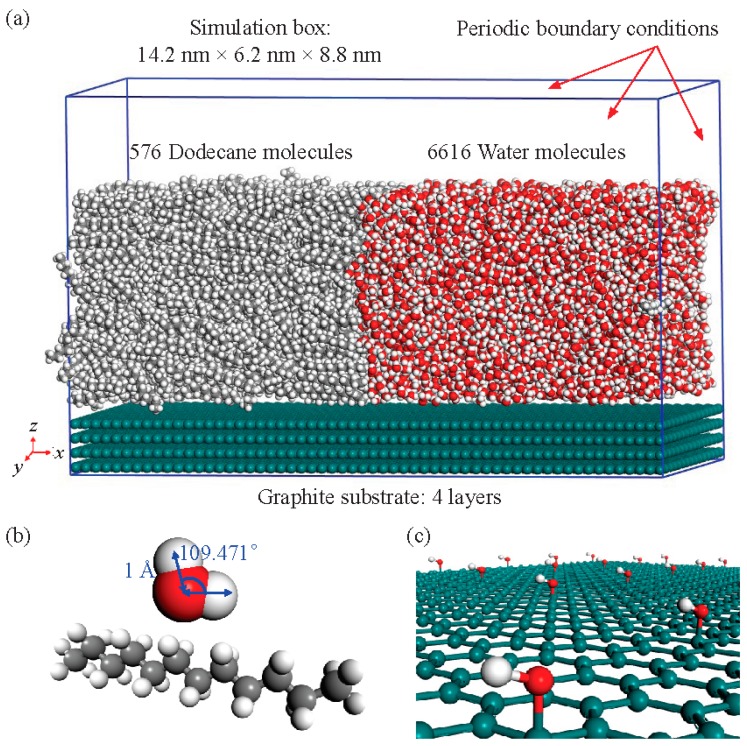
Oil-water-graphite system. (**a**) The final oil-water system (after 100 ps simulation in canonical (NVT) ensemble and 120 ps simulation in isothermal-isobaric (NPT) ensemble) on 4-layer graphite; (**b**) Structure of water and oil molecules; (**c**) Structure of hydroxyl-functionalized graphite surface.

Our MD simulations are implemented through the Forcite module in the Material Studio software (California, CA, USA). We adopt the dodecane (C_12_) to simulate the oil molecules. The simulation system contains 576 C_12_ and 6616 H_2_O of the SPC/E model, which is initially configured in a 16.5 nm × 7 nm × 5.7 nm simulation box. The initial 100 ps simulation allows the system to reach temperature equilibrium in a canonical (NVT) ensemble. Subsequently, the fluid is applied in an isothermal-isobaric (NPT) ensemble, and the system reaches stability in another 120 ps. The system box finally achieves a volume of 14.2 nm × 6 nm × 4.9 nm with a density of 0.871 g cm^−3^ at 1 atm and 298 K. We then arrange the final structure on a piece of 14.2 nm × 6.2 nm four-layer smooth graphite to simulate the solid surface, as shown in Figure 1a. Here, we use a smooth graphite surface, since a rough one would weaken its interaction with water molecules, thus making the surface more hydrophobic. The structures of the oil and water molecules are shown in Figure 1b. For the hydroxylated graphite system, hydroxyls are arranged uniformly on the first layer of the graphite surface to modify the graphite [20]. Figure 1c shows the local configuration of the hydroxylated graphite surface. For different hydroxyl densities, different distances between the hydroxyls are arranged. In this paper, we adopt seven different densities, ranging from 0 to 4.53 nm^−2^. Graphite atoms are fixed during the simulation, while hydroxyl groups can swing on the surface. Periodic boundary conditions are adopted in all directions. A vacant layer with a thickness of 0.2 nm is settled to ensure that the substrate has a weak effect on the fluid from the top side, as shown in Figure 1a. A 100 ps simulation using the Andersen thermostatic condition is then carried out in the NVT ensemble at 298 K to study the properties of the three-phase contact zone in the two systems. The time step is 1 fs. To improve the computational efficiency, only the neighboring atoms within a certain cutoff radius (*r*_cut_) of 1.2 nm are included in the force calculation, since the distant atoms have a negligible contribution. The long-range Coulombic interactions are handled with the three-dimensional Ewald summation.

In our simulation, the interatomic interaction is described by COMPASS force field. The Van der Waals interaction is described by Lennard–Jones 9-6 potential (Equation (1)), and the electronic interaction is described by Coulombic potential (Equation (2)):

(1) $$\phi \left(r_{ij}\right)=\varepsilon_{ij}\left[2{\left(\frac{r_{m,ij}}{r_{ij}}\right)}^{9}-3{\left(\frac{r_{m,ij}}{r_{ij}}\right)}^{6}\right]$$

where *r*_ij_ is the distance between atoms *i* and *j*, and *ε*_ij_ and *r*_m, ij_ are the energy parameter and the length scale, respectively. In Materials Studio, these parameters are determined for a given kind of molecule.

(2) $$\phi_{c}(r_{ij})=\frac{q_{i}q_{j}}{4\pi \varepsilon_{0}r_{ij}}$$

where *q*_i_ and *q*_j_ are the charges of atoms *i* and *j*. *ε*_0_ is the dielectric constant in vacuo.

We first test the contact angle of water droplets wetting the graphite surface to verify the basic method we adopt. Our test system contains 4608 water molecules, which are arranged as a cube on a 16 nm × 16 nm four-layer graphite surface with an initial density of 1 g cm^−3^. The graphite atoms are fixed during the simulation. After the geometry optimization, a simulation is then carried out to reach equilibrium with a time step of 1 fs. The interface markers of the water droplet are obtained by applying the method that Rishi Raj used [21]. The result shows that the contact angle of water droplets on graphite is about 85.9°, which is in good agreement with experimental or other simulation data (80°−90°) [21,22,23,24,25]. Adamson et al. [25] showed a contact angle of 86° by experimental work, and Werder et al. [23] showed 85.5° by MD simulation, which reflects that our results are reasonable.

## 3. Results and Discussion

### 3.1. Molecular Configuration and Microscopic Contact Angle

**Figure 2:**
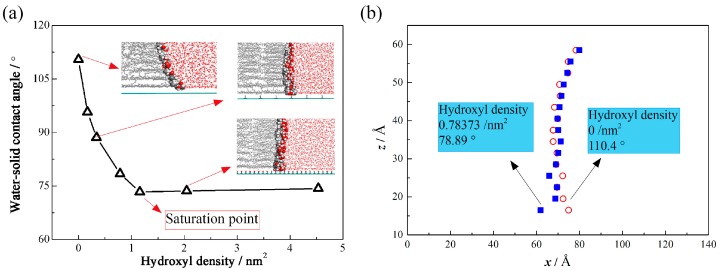
Effect of hydroxyl density on water-solid contact angle. (**a**) Contact angle for graphite system and hydroxylated system with different hydroxyl density (**b**) Oil-water interface points obtained by calculating the water interface and oil interface separately adopting the density method.

Based on the simulation results, the molecule configuration can be visualized. For the condition of the hydroxylated graphite surface, it exhibits a very different configuration of the oil-water interface, especially in the three-phase confluence region near the graphite surface. The contact angle is a parameter that can describe the wettability property of the solid surface. In the oil-water-graphite system, we adopt a microscopic contact angle, which is the angle between the water and graphite [26], to describe the interaction between the surface and liquids. Figure 2a shows the contact angle for the graphite system and the hydroxylated system with different hydroxyl densities (for the figure of the oil-water interface, the exhibit hydroxyl density is 0.78 nm^−2^). This angle is obtained by linear fitting of the oil-water interface points. Figure 2b shows the oil-water interface points, which are obtained by calculating the water interface and oil interface separately using the density method [21]. We then obtain the average points of the water interface and the oil interface. We can see that with the increase of the hydroxyl density, the water-solid contact angle becomes smaller first, and then steady, reflecting the change of the interaction between graphite surface and liquids. For hydroxyls, water molecules play a significant role due to the interaction of hydrogen bonds. We then analyze the adsorption properties of water molecules on the solid surface and the number of hydrogen bonds in systems with different hydroxyl densities, which lets us know more about the mechanism of the effect of the hydroxyl group.

### 3.2. Adsorption Properties of Water Molecules

**Figure 3:**
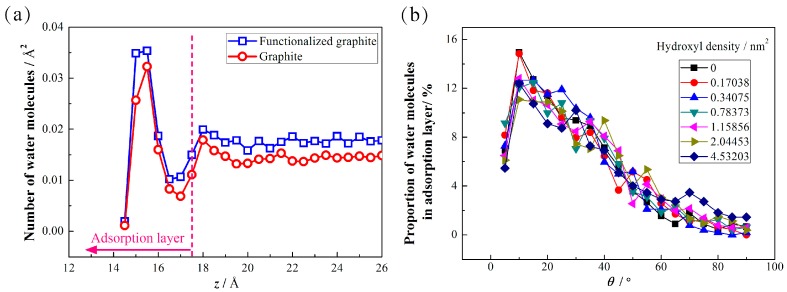
Distribution of water molecules along the *z*-direction. (**a**) Number density of water molecules in two different systems; (**b**) Proportion of water molecules in adsorption layer with different *θ.*

To illustrate the mechanism of the effect of the hydroxyls, the *z*-direction position of water molecules is obtained for gaining adsorption status. An example of 69 hydroxyls is analyzed as a typical case (hydroxylated graphite in Figure 3a). The distribution of water molecules along the *z*-direction is obtained by dividing the simulation region into bins, each Δ*z* = 0.5 Å in size, and the *z* coordinate is stored for H_2_O in the bins. Then, we can obtain the number of water molecules in each bin and calculate the number of water molecules per Å^2^. For the calculation of the area of each bin, we apply the degree of contact angle that was obtained in Figure 2.

Water molecules are adsorbed tightly on the graphite surface due to the strong fluid-solid interaction (Figure 3a). We define an adsorption layer from the graphite surface (*z* = 11.9 Å) to the *x*-*y* plane at *z* = 17.5 Å. For the region outside the adsorption layer, the population distribution fluctuates weakly and exhibits an approximately steady value. There is a peak at *z* = 15.5 Å (0.36 nm from the surface), and for a graphite surface without hydroxyls, the position and the height (ρ/ρ_0_ = 1.979, where ρ is the water density in each bin and ρ_0_ is the water density in bulk) of the peak showed little difference compared to the work by Eslami et al. [27], in which the position was 0.33 nm and ρ/ρ_0_ = 2.2. The hydroxylated graphite system exhibits a more intense peak than the graphite system, which reflects a tighter distribution of the functionalized system, and more water molecules are found in the adsorption layer in the system with the functionalized graphite, which reveals a stronger adsorption force of the functionalized graphite surface on the water molecules. This could reflect the stronger hydrophilicity of the hydroxylated graphite surface.

To know more about the effect of the hydroxyls, we analyze the orientation of water molecules near the surface. Here we define an angle θ. Coordinates of oxygen atoms and hydrogen atoms can be obtained through the MD simulation. Hence, we can calculate the coordinate of the midpoint of the two hydrogen atoms of a water molecule. A line *l* passed through the midpoint and the oxygen atom is obtained. θ is the angle between the line *l* and the graphite surface (*x*-*y* plane). Therefore, angle θ describes the arrangement relationship between water molecules and the graphite surface, and θ = 90° indicates that the water molecule is perpendicular to the *x*-*y* plane. Figure 3b shows the value of θ in the functionalized graphite system and in the graphite system in the adsorption layer, respectively. There is a focus region in the θ range from 10° to 20°, which means that water molecules are more likely to be arranged in this orientation. However, there is no obvious difference for the system with a different hydroxyl density, reflecting that hydroxyls don’t play an important role in inducing water molecules to change their orientation.

To reveal the reason for the orientation property of H_2_O molecules near the surface, adsorption energy of an H_2_O molecule with different θ absorbed on 4-layer graphite is calculated. Here, we consider several different distances between the H_2_O molecule and the graphite surface. The adsorption energy is calculated by the following equation:

(3) $$E=\frac{E_{\mathrm{tot}}-E_{\mathrm{gra}}-nE_{i}}{n}$$

where *E*_tot_ is the total energy of the system after the molecules are adsorbed on the surface, and *E*_gra_ is the energy of the surface, *n* is the number of the molecules, *E*_t_ is the energy of one molecule. Positive values indicate endothermic conditions, and negative values indicate exothermic conditions. Under endothermic conditions, a larger value means harder adsorption on the surface. Under the exothermic conditions, a larger value of E means easier adsorption on the surface.

**Figure 4:**
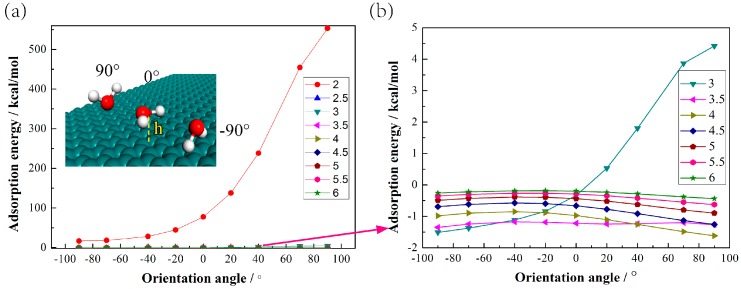
Adsorption energy for a single water molecule. (**a**) Arrangement of single water molecule in adsorption layer on graphite surface and adsorption energy with different distances h and orientation angles; (**b**) Details of adsorption energy for single water molecule for distances larger than 3 Å.

We perform some MD simulation work, and find that when the distance between the water molecule and the graphite surface is 3 Å (water molecule in the adsorption layer), the exothermic process happens when the hydrogen atom is toward the surface, which means hydrogen atoms tend inward for single water molecules, as Figure 4 shows. But when the distance between the water molecule and the graphite is larger than 3 Å, there is little change of the adsorption energy, and we can also see the trend that a single water molecule seems to find it easier to be perpendicular to the surface. In this case, the position of molecules contributes more than the orientation, since it exhibits bulk-like orientation, which is qualitatively the same as the work by Eslami et al, [28]. These results provide additional details on the orientation of water molecules, in addition to what is shown in Figure 3b. Also, water molecules can display very different behaviors from each other; a net of hydrogen bonds is formed to make the adsorption more stable [19,29], which causes the final results of the orientation in Figure 3b.

### 3.3. Number of Hydrogen Bonds

To further explain the mechanism of the effect of hydroxyls on the wettability of the graphite surface, a number of hydrogen bonds are analyzed. *R*_OH_ in neighboring water molecules is widely used to express the length of the hydrogen bond. The criteria for the existence of a hydrogen bond are the length of hydrogen bond and the value of the angle H–O…H [30]. Here, we assume that *R*_OH_ is less than 3.5 Å and that the angle H–O…H is larger than 130 degrees [27]. We find that the number of hydrogen bonds formed between water molecules in the adsorption layer increases with the increase in hydroxyl density, which shows a different trend from that with the contact angle. So, the hydrogen bonds formed between water molecules may not be the main factor governing the change of the contact angle.

**Figure 5:**
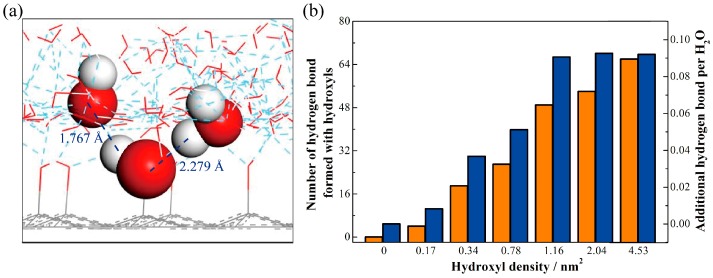
Details of hydrogen bonds formed between hydroxyls and water molecules. (**a**) Microscopic view of hydrogen bond formed between hydroxyls and water molecules; (**b**) Number of hydrogen bonds formed by hydroxyls (orange histogram) and additional hydrogen bonds per water molecule in adsorption layer (blue histogram).

We then calculate the number of hydrogen bonds that the hydroxyls form; the microscopic view is shown in Figure 5a. For a hydroxyl density of 0.78 nm^−2^, 27 hydrogen bonds are formed between hydroxyls and water molecules. Water molecules at the solid-liquid interface are governed by the intermolecular force between the solid wall and the liquid bulk. As a kind of intermolecular interaction, the hydrogen bond has a stronger attraction force than the van der Waals interaction. Thus, the hydrogen bond has a significant attraction to the water molecules at the solid-liquid interface. Hydrogen bonds between hydroxyls and water molecules help to attract more water molecules close to the solid surface. Therefore, a stronger hydrophilicity is formed on the hydroxylated graphite surface. Figure 5b displays the number of hydrogen bonds formed between hydroxyls and water molecules in the adsorption layer. These results indicate that the hydroxyls on the graphite surface induce the water molecules to form more hydrogen bonds. These hydrogen bonds between hydroxyls and the water molecules in the bulk give an additional adsorption force to the water phase; thus, more water molecules exist in the adsorption layer. The number of hydrogen bonds formed between hydroxyls and water molecules increases with the increase in hydroxyl density. However, the additional hydrogen bond generated by hydroxyls per water molecule remains almost the same when the hydroxyl density exceeds a critical value—which we call the saturation point—at a density of 1.16 nm^−2^. This is due to space and orientation effects, which limit the water molecules’ distribution, and thus impose a maximum number for hydrogen bond formation. All of the evidence shows that the hydrogen bond network formed by water molecules in the adsorption layer is stable enough. When more hydroxyls appear, the intensity of interaction of the hydroxyl and water molecules is not strong enough to destroy the main hydrogen bond network. Therefore, the number of hydrogen bonds per water molecule stabilizes.

## 4. Conclusions

In this paper, the hydroxylated graphite surface is generated as a hydrophilic oleophobic material for the application of oil-water separation, and the effects of hydroxyl density on the surface wettability are studied in oil-water-graphite three-phase systems via MD simulation. Results show that hydroxyls on the graphite surface cause a smaller microscopic contact angle on the water side. Notably, a critical hydroxyl density is found, in excess of which the contact angle is almost unchanged. We attribute these phenomena to the hydrogen bonds formed between hydroxyls and water molecules. Hydroxyls can form hydrogen bonds with water molecules, which cause stronger interaction between water molecules and the solid surface and accordingly lower the contact angle. With increasing hydroxyl density, the contact angle gradually reaches an equilibrium value owing to the limited density of the hydrogen bonds between the hydroxyls and water molecules in the adsorption layer. With an increase in hydroxyls, the hydrogen bond network becomes more stable, leading to an increased difficulty in the formation of hydrogen bonds between hydroxyls and water molecules. Consequently, this work is helpful, and can be instructive for material design in the field of oil-water separation.
